# Ultrathin Descemet stripping automated endothelial keratoplasty versus Descemet membrane endothelial keratoplasty: a fellow-eye comparison

**DOI:** 10.1186/s40662-020-00191-6

**Published:** 2020-05-06

**Authors:** Rita Mencucci, Eleonora Favuzza, Elisa Marziali, Michela Cennamo, Cosimo Mazzotta, Ersilia Lucenteforte, Gianni Virgili, Stanislao Rizzo

**Affiliations:** 1 Eye Clinic, Department of Neuroscience, Psychology, Pharmacology and Child Health (NEUROFARBA), University of Florence, Eye Clinic, Largo Brambilla 3, 50134 Florence, Italy; 2 Department of Medicine, Surgery and Neurosciences, Ophthalmology Unit, Siena University, Siena, Italy; 3 Siena Crosslinking Center, Siena, Italy; 4 Department of Clinical and Experimental medicine, University of Pisa, Pisa, Italy

**Keywords:** DMEK, UT-DSAEK, DSAEK, Descemet stripping automated endothelial keratoplasty, Ultra-thin Descemet stripping automated endothelial keratoplasty, Descemet membrane endothelial keratoplasty

## Abstract

**Background:**

To compare the visual outcome and patients’ satisfaction after ultrathin Descemet stripping automated endothelial keratoplasty (UT-DSAEK) and Descemet membrane endothelial keratoplasty (DMEK) performed on fellow eyes of the same patients.

**Methods:**

In this retrospective study, the records of 18 pseudophakic patients affected by Fuchs endothelial dystrophy who underwent DMEK in one eye and UT-DSAEK in the fellow eye were reviewed. Best corrected visual acuity (BCVA), corneal pachymetry, keratometry, corneal aberrations, photopic and mesopic contrast sensitivity, and endothelial cell counts measured 12 months after surgery in either eye were analyzed and compared. The results of a satisfaction questionnaire were also reviewed.

**Results:**

Twelve months after surgery, BCVA was not significantly different in UT-DSAEK and DMEK eyes (0.10 ± 0.04 and 0.07 ± 0.07 logMAR, respectively); at both 4- and 6 mm optical zones total and posterior corneal higher order aberrations (HOAs), posterior astigmatism and total coma were significantly lower after DMEK; BCVA in both groups was significantly correlated mainly with anterior corneal aberrations; contrast sensitivity was higher after DMEK especially in mesopic conditions and at medium spatial frequencies; the endothelial cell density was similar, although slightly higher in the UT-DSAEK group (*p* = 0.10). The satisfaction questionnaire showed that although patients were highly satisfied from both procedures, more than half of them preferred DMEK and reported a more comfortable and quicker postoperative recovery.

**Conclusions:**

DMEK and UT-DSAEK showed no evidence of difference in terms of postoperative BCVA, although DMEK had a better performance in terms of contrast sensitivity, posterior corneal aberrations and overall patient satisfaction.

## Background

In the past decade, endothelial keratoplasty has become the gold standard for the treatment of endothelial dysfunctions, such as Fuchs endothelial dystrophy or pseudophakic bullous keratopathy [1]. More recently, a new procedure that allows us to selectively transplant only the Descemet membrane and the endothelium, Descemet membrane endothelial keratoplasty (DMEK), has been developed [2]; this technique is a refinement of the Descemet stripping automated endothelial keratoplasty (DSAEK), where a relatively thick graft composed of endothelium, Descemet membrane, and a variable portion of posterior stroma is transplanted.

Due to its quicker postoperative recovery and similar or better visual outcome and lower rejection rates, DMEK has rapidly gained popularity even though its widespread adoption is still limited by the difficult and less predictable surgical technique that prevents its use in complicated cases, and by its higher rates of postoperative rebubbling [3–5].

To the best of our knowledge, the majority of published studies and metanalysis comparing DSAEK and DMEK outcomes [1, 5–10] take into consideration only DSAEK grafts which are thicker than 130 μm.

Even though the debate on the relationship between graft thickness and DSAEK visual outcome is still ongoing and is based on contradictory results [11, 12], Neff et al. [13] were the first to suggest that DSAEK grafts thinner than 130 μm [ultrathin DSAEK (UT-DSAEK)] may lead to postoperative visual outcomes that are better than conventional DSAEK and comparable to DMEK. These outcomes were further supported by a large interventional case series [14] and by a recent randomized controlled clinical trial [4]. Thus, due to the higher demand and thanks to the refinement and standardization of graft cutting techniques [3, 14, 15], eye banks nowadays are providing surgeons with thinner grafts, often thinner than 100 μm (ultrathin) or 50 μm (nanothin) [3, 16]. The increasing availability of ultrathin grafts has led to a randomized controlled clinical study [17] which found a higher visual outcome after DMEK compared to UT-DSAEK.

In this context, the aim of our study was to retrospectively compare the visual outcomes, contrast sensitivity, corneal keratometry and aberrations, endothelial cell density (ECD) and satisfaction in patients affected by Fuchs endothelial dystrophy who underwent UT-DSAEK in one eye and DMEK in the fellow eye.

## Methods

In this retrospective study, the records of 18 pseudophakic patients (implanted with a spherical monofocal hydrophobic acrylic IOL, SA60AT, Alcon, Fort Worth, Texas, USA) affected by Fuchs endothelial dystrophy who underwent DMEK in one eye and UT-DSAEK in the fellow eye were reviewed.

All surgical procedures were performed by the same experienced surgeon (R.M.) between January 2015 and June 2017. The procedures were carried out at the Eye Clinic, Department of Neuroscience, Psychology, Pharmacology and Child Health (NEUROFARBA), University of Florence, Italy.

Only patients who had at least 12 months of postoperative follow-up were included. In all patients UT-DSAEK was performed in the first eye and DMEK in the second eye: the first eye was operated when the surgeon (R.M.) had limited experience with DMEK and preferred UT-DSAEK, the second eye was when the surgeon became more confident in the new technique and started to perform DMEK in all uncomplicated cases.

One patient who experienced severe postoperative complications after DMEK (pupillary block and total graft detachment, corneal decompensation that did not recover after air injection and was successfully managed with UT-DSAEK graft implantation) was excluded. Two patients affected by severe macular degeneration with low visual potential were also excluded.

This retrospective observational study received the approval of the local Ethics Committee and was conducted in compliance with the tenets of the Declaration of Helsinki. All patients were informed about the study and provided consent.

At the preoperative visit, best corrected visual acuity (BCVA, logMAR), slit-lamp examination, applanation tonometry (Goldmann applanation tonometer, Haag Streit, Bern, Switzerland), ocular fundus examination, ECD (Perseus, CSO, Italy) and corneal pachymetry (Sirius tomographer, CSO, Italy) were performed.

The patients were examined at 1 and 10 days and at 1,3, 6, and 12 months after surgery. At the 12-month visit, in addition to the previously mentioned measurements, corneal aberrations at 4 and 6 mm optical zones (Sirius, CSO, Italy), graft thickness [spectral-domain anterior-segment optical coherence tomography (AS-OCT), RT-Vue OCT, Optovue Inc., Fremont, CA, USA] and contrast sensitivity were measured.

Graft thickness was measured using AS-OCT on a horizontal cross-sectional image obtained at the anterior corneal vertex, using the software-imbedded tool. Central thickness of the graft was measured at the corneal vertex, the peripheral thickness at the temporal and nasal sides at a distance of 3 mm from the vertex. The mean of the temporal and nasal thickness for each patient was then recorded.

Distance contrast sensitivity was analyzed under photopic and mesopic conditions (85 and 3 cd/m, respectively) at six spatial frequencies (A, 1.5 cycles per degree, cpd; B, 3 cpd; C, 6 cpd; D, 12 cpd; E, 18 cpd) using the Optec 6500 Vision Tester (Stereo Optical Co., Inc., Chicago, USA) and was compared to the physiologic contrast sensitivity range of the measuring device for normal patients of a similar age [18].

A questionnaire grading the patient’s satisfaction with surgery for right and left eye on a scale of 1 to 6 including the following questions was administered at the last follow up visit (Goldich et al. [19], slightly modified): How is your vision? (1 = very bad, 2 = bad, 3 = fine, 4 = good, 5 = very good, 6 = excellent). Compare the post-operative period for the respective eye in terms of comfort. (1 = very comfortable, 6 = very uncomfortable). How long did it take to resume normal activities (i.e., go back to work)? Please rate your level of satisfaction from surgery (1 = least satisfied, 6 = most satisfied). Having undergone the two types of surgery, which did you prefer? Even though this questionnaire has not been validated, it is the only currently available tool suitable for fellow-up eye studies.

Demographic characteristics, preoperative BCVA and corneal pachymetry, donor and graft characteristics and the aforementioned 12-month postoperative parameters were reviewed and analyzed for this retrospective study.

### Surgical techniques

All procedures were performed under monitored anesthesia with peribulbar block. Posterior lamellar grafts were supplied by the Eye Bank of Lucca (Italy), after being cut by a microkeratome with a 350-μm head (Moria SA, Antony, France) (DSAEK grafts) or being stripped and placed on their sclerocorneal support (DMEK grafts). They were trephined by the surgeon to the desired diameter using a Hessburg-Barron donor corneal punch (Barron Precision Instruments, LLC, Grand Blanc, Michigan USA); DMEK grafts were left on their natural support immersed in 0.06% trypan blue dye (Vision blue; D.O.R.C). All grafts had an endothelial cell count of at least 2500 cells/mm^2^. The graft thickness provided by the Bank was recorded.

The epithelium of the recipient was marked with a trephine in order to guide the subsequent descemetorhexis and to allow the correct positioning and perfect centering of the transplanted donor flap. The anterior chamber (AC) of the eye was then entered through a clear corneal incision, and an anterior chamber maintainer (ACM) was used in order to prevent an anterior chamber collapse.

The endothelium and the Descemet membrane were stripped from the central 8.5 to 9 mm diameter using the inverted Price-Sinskey hook, along the epithelial reference line for about 45° or 90°. The removed flap was exposed on the anterior surface of the receiver’s cornea to verify its integrity.

In DSAEK surgery, the rolled donor’s endothelial graft was inserted using a Busin glide through a 4 mm clear corneal incision (Moria Inc., Antony, France) and a small air bubble was injected to lift the graft. After centering the graft, the anterior chamber was completely filled with an air bubble to allow the perfect adherence of the donor flap to the receiving tissue [20].

DMEK surgery was performed following the “no-touch” technique. The trephined DMEK graft was carefully detached from the surrounding DM, immersed in sterile balanced salt solution and aspirated into the transparent glass cartridge of a specific injector (E. Janach S.R.L., Como, Italy). The rolled graft was injected into the AC with slow and continuous pressure through the main incision (about 3 mm). The graft was then unfolded and positioned using the Tap-tap technique and Dirisamer technique, and after ensuring the correct orientation and centration, it was pressed against the recipient stroma by injecting air underneath.

Patients were instructed to keep a supine position after surgery until the air in the AC was completely reabsorbed. In the case of a pupillary block or ocular hypertension, a small quantity of air was released using a slit lamp. The postoperative treatment for both groups was a topical antibiotic given 4 times a day for the first 3 weeks, and dexamethasone eye drops 4 times a day for the first month. The topical steroid was tapered down to one drop every other day during a 1-year period.

### Statistical analysis

Data are presented as mean ± standard deviation (SD). After the normality of distribution of values within each data set of continuous variables had been checked with the Kolmogorov-Smirnov test, either paired two-tailed Student’s t test (parametric) or the Wilcoxon signed rank test (non-parametric) was used to assess differences. Correlations between BCVA and corneal aberrations were analyzed by Spearman’s test.

In order to make inference on the differences in contrast sensitivity between DMEK and DSAEK, we fitted a single statistical model using random effects linear mixed modelling with CS as a dependent variable and procedure type and contrast frequency as covariates, in which we tested for interaction between procedure type and, separately, light conditions and frequency. We fitted separate models for the photopic and mesopic conditions. Analyses were performed using SPSS and Stata 14.1 software (StataCorp, College Station, TX). Values of *p* < 0.05 were considered as statistically significant.

## Results

In this study, we included 36 eyes of 18 pseudophakic patients (16 females and 2 males) affected by bilateral Fuchs dystrophy, with a mean age of 73.5 ± 7.93 years. Patients underwent UT-DSAEK in one eye and after an average of 6 months (6.3 ± 1.2 months) DMEK in their fellow eye.

The preoperative characteristics of patients are shown in Table 1. The differences between the two groups in preoperative BCVA (log MAR) (*p* = 0.10) and preoperative pachymetry (μm) (*p* = 0.14) were not statistically significant. Table 1 also reports donor and graft characteristics in both groups. The preoperative mean thickness of the UT-DSAEK graft was 80.33 ± 20.52 μm. The mean age of donors, the endothelial cell count (measured by the eye bank after tissue processing) and the diameter of the graft were not significantly different.

**Table 1:** Preoperative characteristics of patients and the transplanted grafts

	UT-DSAEK	DMEK	p
Preoperative BCVA (log MAR)	0.60 ± 0.29	0.51 ± 0.11	0.10
Preoperative pachymetry (μm)	618.78 ± 39.41	629.28 ± 38.64	0.14
Donor age (y)	67.17 ± 6.27	69.56 ± 9.82	0.45
Graft ECD (cell/mm2)	2700.00 ± 59.41	2625.56 ± 124.58	0.06
Graft central thickness (μm)	80.33 ± 20.52	–	–
Diameter of the graft (μm)	7.99 ± 0.18	7.88 ± 0.13	0.10

*P* value was assessed by the Paired-t test for BCVA, pachymetry and donor age, and by the Wilcoxon signed rank test (non-parametric) for graft ECD and diameter. *UT-DSAEK*= ultra-thin Descemet stripping automated endothelial keratoplasty; DMEK= Descemet membrane endothelial keratoplasty; *BCVA=* best corrected visual acuity; *logMA*R= logarithm of the minimal angle of resolution; y= years; *ECD=* endothelial cell density. Data are presented as mean ± standard deviation (SD)

No patient showed iris damage or had undergone previous ocular surgery other than uncomplicated phacoemulsification with posterior chamber IOL implantation or had very deep anterior chamber in either eye.

One eye in the DSAEK group and three eyes in the DMEK group experienced early postoperative peripheral partial graft detachment, which was successfully managed with air injection in the anterior chamber within 1 week from surgery. No intraocular pressure rise or graft failures or rejections were observed in this retrospective study. No patients were reported to have significant posterior capsular opacification.

### Postoperative visual acuity, refraction and endothelial cell density

BCVA significantly improved 12 months after UT-DSAEK and DMEK (*p* < 0.001 and *p* < 0.001, respectively, Wilcoxon signed rank test) without any statistically significant difference between the two groups (*p* = 0.24, Wilcoxon signed rank test, Table 2). Using a paired t-test, a difference of 0.023 logMAR (about 1 letter) favoring DMEK was found, but clinically important differences (2.5 letters or more) were unlikely (95% CI: − 0.003 to 0.049, *p* = 0.07). The objective and subjective refraction and the spherical equivalent did not significantly differ between UT-DSAEK and DMEK eyes, except for the objective sphere which was significantly lower after DMEK (*p* < 0.001). The keratometric values evaluated by the Sirius Tomographer (CSO, Italy) were not significantly different between groups.

**Table 2:** Postoperative results at the 12-month follow-up after UT-DSAEK and DMEK

	UT-DSAEK	DMEK	p
BCVA (logMAR)	0.10 ± 0.04	0.07 ± 0.07	0.24
Objective refraction (sphere, D)	0.92 ± 0.40	0.56 ± 0.17	0.001**
Objective refraction (cyl, D)	−1.12 ± 0.55	−1.00 ± 0.34	0.45
Subjective refraction (sphere, D)	0.56 ± 0.47	0.29 ± 0.25	0.07
Subjective refraction (cyl, D)	−0.62 ± 0.67	−0.62 ± 0.52	0.82
Spherical Equivalent (D)	0.25 ± 0.39	− 0.01 ± 0.33	0.07
Sim K1 (D)	43.42 ± 0.67	43.51 ± 0.96	0.14
Sim K2 (D)	44.39 ± 0.85	44.30 ± 0.97	0.77
Avg (D)	43.90 ± 0.75	43.91 ± 0.95	0.67
Cyl total (D)	−0.98 ± 0.27	−0.79 ± 0.30	0.11
Corneal pachymetry (μm)	570.38 ± 21.96	516.29 ± 33.52	< 0.001**
Graft central thickness (μm)	77.85 ± 22.02	–	–
Graft peripheral thickness (μm)	107.46 ± 28.32	–	–
ECD (cell/mm^2^)	1772.62 ± 185.59	1590.94 ± 136.87	0.10

*P* value was assessed by the Wilcoxon signed rank test (non-parametric); *UT-DSAEK=* ultra-thin Descemet stripping automated endothelial keratoplasty; *DMEK=* Descemet membrane endothelial keratoplasty; *BCVA=* best corrected visual acuity; *logMAR=* logarithm of the minimal angle of resolution; *SIMK=* K-value of simulated keratometry; *Avg=* average; *Cyl=* cylinder; *D=* diopters; *ECD=* endothelial cell density. Data are presented as mean ± standard deviation (SD). **p < 0.01

The ECD after the 12-month follow-up was similar, although slightly higher in the UT-DSAEK group (*p* = 0.10) and as expected, the corneal thickness was lower (*p* < 0.001) in the DMEK group (Table 2).

### Corneal aberrations

Tables 3 and 4 present postoperative corneal aberrations as evaluated by the Sirius Tomographer. Total and posterior corneal higher order aberrations (HOAs), posterior astigmatism and total coma were significantly lower after DMEK than UT-DSAEK at both 4 and 6 mm optical zones. The posterior coma was significantly lower in DMEK only at the 4 mm optical zone. The total and anterior corneal astigmatism were significantly lower in the DMEK group only at the 6 mm optical zone. The spherical aberration was similar between groups.

**Table 3:** Corneal aberrations (μm) at a 4 mm optical zone 12 months after surgery

	UT-DSAEK	DMEK	p
HOAs total	0.38 ± 0.09	0.29 ± 0.10	0.01*
HOAs front	0.27 ± 0.08	0.25 ± 0.09	0.89
HOAs back	0.24 ± 0.13	0.13 ± 0.04	0.001**
Astigmatism total	0.45 ± 0.26	0.41 ± 0.17	0.97
Astigmatism front	0.40 ± 0.15	0.35 ± 0.14	0.23
Astigmatism back	0.22 ± 0.07	0.16 ± 0.06	0.01*
Coma total	0.28 ± 0.06	0.20 ± 0.07	< 0.001**
Coma front	0.20 ± 0.06	0.16 ± 0.06	0.20
Coma back	0.12 ± 0.08	0.05 ± 0.02	0.003**
Spherical aberration total	0.07 ± 0.04	0.05 ± 0.03	0.16
Spherical aberration front	0.07 ± 0.04	0.06 ± 0.02	0.40
Spherical aberration back	0.04 ± 0.03	0.02 ± 0.01	0.18

*P* value was assessed by the Wilcoxon signed rank test (non-parametric); *UT-DSAEK=* ultra-thin Descemet stripping automated endothelial keratoplasty; *DMEK=* Descemet membrane endothelial keratoplasty; *HOAs=* high-order aberrations; *p < 0.05; **p < 0.01. Data are presented as mean ± standard deviation (SD). All values are in micrometers

**Table 4:** Corneal aberrations (μm) at a 6 mm optical zone 12 months after surgery

	UT-DSAEK	DMEK	p
HOAs total	0.88 ± 0.20	0.58 ± 0.15	< 0.001**
HOAs front	0.76 ± 0.16	0.63 ± 0.19	0.11
HOAs back	0.43 ± 0.16	0.23 ± 0.07	< 0.001**
Astigmatism total	1.08 ± 0.43	0.63 ± 0.34	0.003**
Astigmatism front	0.95 ± 0.27	0.62 ± 0.24	0.005**
Astigmatism back	0.41 ± 0.17	0.20 ± 0.11	< 0.001**
Coma total	0.55 ± 0.10	0.45 ± 0.26	0.04*
Coma front	0.44 ± 0.16	0.45 ± 0.24	0.87
Coma back	0.20 ± 0.13	0.14 ± 0.10	0.06
Spherical aberration total	0.24 ± 0.14	0.24 ± 0.06	0.87
Spherical aberration front	0.26 ± 0.08	0.25 ± 0.04	0.89
Spherical aberration back	0.09 ± 0.07	0.18 ± 0.28	0.76

*P* value was assessed by the Wilcoxon signed rank test (non-parametric); *UT-DSAEK=* ultrathin Descemet stripping automated endothelial keratoplasty; *DMEK=* Descemet membrane endothelial keratoplasty; *HOAs=* high order aberrations; *p < 0.05; **p < 0.01. Data are presented as mean ± standard deviation (SD). All values are in micrometers

### Correlations between postoperative BCVA and corneal aberrations

Spearman’s correlation coefficients are reported in Table 5. BCVA 12 months after UT-DSAEK was significantly correlated with anterior HOAs, anterior astigmatism, total and anterior coma and anterior spherical aberration at a 4 mm optical zone; in DMEK eyes postoperative BCVA was correlated with total and anterior HOAs and total and anterior astigmatism at a 4 mm optical zone. Correlations between BCVA and aberrations at a 6 mm optical zone are reported in Table 5. All the significant correlations (except the posterior coma at a 6 mm optical zone) were positive (e.g., the higher the aberration, the higher the BCVA logMAR value and the lower the visual acuity). The posterior coma at a 6 mm optical zone was negatively correlated to the BCVA (the higher the aberration, the lower the BCVA logMAR value and the higher the visual acuity) but the level of significance was only at 0.030.

**Table 5:** Correlations between BCVA and corneal aberrations at 4- and 6 mm optical zones 12 months after surgery

	UT-DSAEK 4.0 mm	DMEK 4.0 mm	UT-DSAEK 6.0 mm	DMEK 6.0 mm
	r	r	r	r
HOAs total	− 0.091	0.618**	0.213	0.448
HOAs front	0.647**	0.912**	0.334	0.500*
HOAs back	−0.091	− 0.059	0.334	0.431
Astigmatism total	−0.030	0.529*	0.030	0.059
Astigmatism front	0.698**	0.529*	0.577*	0.059
Astigmatism back	−0.213	−0.309	−0.091	− 0.412
Coma total	0.493*	0.418	0.954**	0.409
Coma front	0.556*	0.322	0.880**	0.413
Coma back	−0.273	0.448	−0.516*	0.344
Spherical aberration total	0.375	0.446	−0.395	0.425
Spherical aberration front	0.638**	0.435	−0.455	0.224
Spherical aberration back	0.063	−0.405	−0.395	0.224

Spearman’s correlation coefficients (r) are reported. *UT-DSAEK=* ultrathin Descemet stripping automated endothelial keratoplasty; *DMEK=* Descemet membrane endothelial keratoplasty; *HOAs=* high order aberrations; *p < 0.05; **p < 0.01

### Contrast sensitivity

**Fig. 1:**
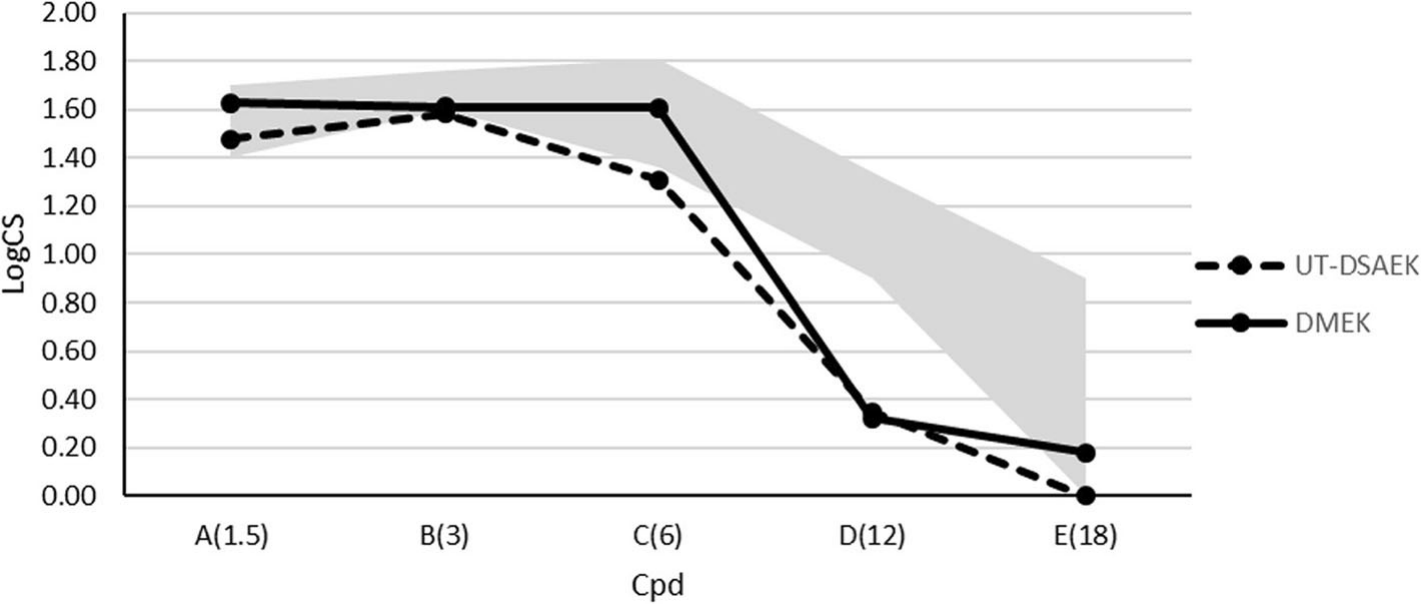
Contrast sensitivity (logCS) measured with the Optec 6500 Vision Tester under photopic conditions at different spatial frequencies (cycles per degree) at 12 months postoperative. The gray area represents the normal range of similar age subjects [18]

**Fig. 2:**
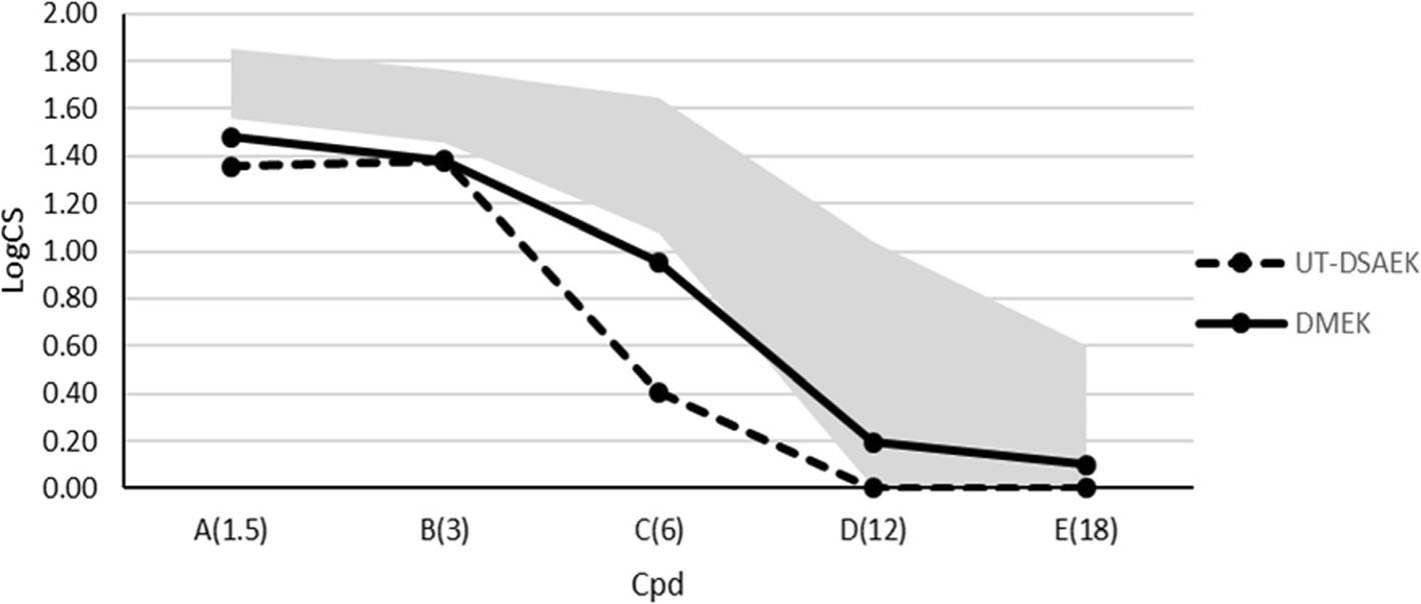
Contrast sensitivity (logCS) under mesopic conditions measured with the Optec 6500 Vision Tester at different spatial frequencies (cycles per degree) 12 months after surgery. The gray area represents the normal range of similar age subjects [18]

Contrast sensitivity results were reported in Figs. 1 and 2; they were at the lowest limit or below the physiological contrast sensitivity range, especially in mesopic conditions, in both groups, compared to the linear model of normal patients of similar age (Fig. 2).

Using linear mixed modelling, we found no overall interaction between procedure and spatial frequency in the photopic condition (*p* = 0.354), where only frequency C (6 cpd) showed a borderline difference between procedures (*p* = 0.014). Therefore, we averaged the differences across frequencies and found an average difference between UT-DSAEK and DMEK eyes of 0.12 logCS favoring DMEK (*p* = 0.022).

In mesopic conditions we found a significant overall interaction between procedure and frequency (*p* = 0.017), a heterogeneity which, again, was mainly related to a much larger difference in frequency C (6 cpd) (*p* < 0.001). By averaging the differences across frequencies, we found that DMEK outperformed DSAEK by 0.20 logCS (*p* < 0.001) more than in photopic conditions.

### Satisfaction questionnaire

Patients were asked to evaluate visual outcomes on a scale of 1–6 (1 = very bad, 6 = excellent): for the UT-DSAEK eye, the mean rate was 4.68 ± 0.49, for the DMEK eye, the score was 5.00 ± 0.84 (*p* = 0.031, Wilcoxon signed rank test), which significantly favored DMEK. Overall, patients were highly satisfied with their vision in both eyes even though 33% reported good and 67% very good vision in the UT- DSAEK eye, whereas in the DMEK eye, 33% reported good vision, 33% very good vision and a further 33% excellent vision.

The majority of patients (83.4%) reported a very comfortable postoperative period after DMEK, while only half of them after UT-DSAEK; using a scale from 1 to 6 (1 = very comfortable, 6 = very uncomfortable), the mean score was 1.50 ± 0.51 after UT-DSAEK and 1.17 ± 0.38 after DMEK (p = 0.031).

The mean recovery time to resume normal activities (question 3) was 20.83 ± 13.09 days after UT-DSAEK and significantly lower, 14.00 ± 9.41 days, after DMEK (*p* < 0.001).

Patients were highly satisfied with both procedures (question 4): in both UT-DSAEK and DMEK eyes, the score was 6 in 83.4% of patients (on a scale from 1 = lest satisfied to 6 = most satisfied), and the mean was 5.67 ± 0.67 after UT-DSAEK and 5.83 ± 0.38 after DMEK (*p* = 0.344). Nevertheless, 66.7% of patients (12 out of 18) preferred DMEK to UT-DSAEK (question 5).

## Discussion

DMEK and DSAEK are the two most widely performed endokeratoplasty techniques for the treatment of endothelial dysfunctions. Even though there is evidence that DMEK may give equal or better results than “conventional” DSAEK (with grafts thicker than 130 μm and a faster recovery time [1, 5–10], few studies have directly compared the visual outcomes between UT-DSAEK and DMEK [16, 17, 21, 22].

While Bhandari et al. [21] and Chamberlain et al. [17] found better visual outcomes after DMEK compared to UT-DSAEK, Tourabaly et al. [22] found similar BCVA between DMEK, UT-DSAEK and NT-DSAEK and Kurji et al. [16] between DMEK and NT-DSAEK.

In our study, comparing the outcomes of UT-DSAEK and DMEK performed on fellow eyes, we found similar best corrected visual acuity at 12 postoperative months (0.10 ± 0.04 logMAR in UT-DSAEK, 0.07 ± 0.07 logMAR in DMEK eyes, *p* = 0.24). Our results in logMAR were better than those reported in the only published contralateral-eye comparison between UT-DSAEK (0.34 ± 0.1 logMAR, mean graft thickness 91.1 ± 10.1 μm) and DMEK (0.21 ± 0.12 logMAR) [21], which found a statistically significant difference between the two techniques favoring DMEK. Our results are more similar to a recent randomized trial, the DETECT study [17] that found a visual acuity of 0.04 ± 0.12 logMAR in 25 eyes subjected to DMEK and 0.16 ± 0.18 logMAR in 25 eyes subjected to UT-DSAEK (central graft thickness 73 ± 12 μm), although with a significant difference between groups; conversely, the retrospective study of Tourabaly et al. [22] found a mean postoperative BCVA of 0.09 logMAR in DMEK eyes (*n* = 30) and 0.17 logMAR in UT-DSAEK eyes (*n* = 30), without significant differences between groups.

Regarding the previously reported quicker postoperative recovery after DMEK compared to DSAEK [17, 22, 23], in our study we could not evaluate this parameter as only 12-month follow-up results were analyzed. Further larger or longer prospective studies comparing UT-DSAEK and DMEK visual outcomes are necessary to more precisely assess the differences.

The postoperative endothelial cell count, while not showing statistically significant differences in the two groups, was better after UT-DSAEK than DMEK. These results, corresponding to a mean ECD loss of 34.83 and 38.01% respectively and comparable to other studies [23], are probably caused by the increased handling of DMEK tissue during surgery. Even though none of the included patients had intraoperative complications, the higher number of early partial graft detachment (1 in the UT-DSAEK group and 3 in the DMEK group), the higher complexity of the DMEK technique and the relatively lower experience of the surgeon with DMEK (the analyzed cases were within the surgeon’s first hundred) may explain our results.

It has been pointed out that the visual performance of patients undergoing EK can be influenced by multiple factors, e.g., the duration of the disease, HOA, haze-related light reflection phenomena, parallelism and irregularities of the host-donor interface [24, 25]. In our study, posterior corneal aberrations such as HOAs, astigmatism and coma were significantly lower after DMEK than UT-DSAEK, while the anterior aberrations did not significantly differ. Nevertheless, postoperative visual acuity was significantly correlated (the higher the aberrations, the lower the visual acuity) mainly with total or anterior aberrations such as HOAs and astigmatism in the DMEK eyes, and with HOAs, astigmatism, coma and spherical aberration in the UT-DSAEK eyes. Our results are generally in line with studies comparing DSAEK and DMEK which found higher posterior aberrations in DSAEK eyes [26–31]. In fact, the stromal lamella present in DSAEK grafts seems to be responsible for posterior astigmatism, hyperopic shift and HOAs [26, 27]. Despite the continuous improvement in DSAEK graft preparation and regularity, a difference in posterior corneal aberrations seems to still be present even with thinner grafts [28]. Only two studies about aberrations after UT-DSAEK and DMEK have been published up till now [22, 28]: while the retrospective study of Tourabaly et al. [22], which evaluated the total ocular aberrations, did not find any difference, the randomized controlled prospective study DETECT [28] found significantly higher posterior corneal HOAs, coma and trefoil at the 4 mm optical zone and significantly higher posterior corneal coma, astigmatism, tetrafoil and HOAs at the 6 mm optical zone 12 months after UT-DSAEK compared to DMEK. In our study, however, contrary to what was reported by Duggan et al. [28], total HOAs and total coma were higher after UT-DSAEK than after DMEK at both 4 and 6 mm optical zones.

Despite these differences between the two groups in posterior corneal aberrations, our study’s visual acuity seems to be more influenced by anterior corneal aberrations; this would confirm the importance of the anterior corneal changes (haze or fibrotic changes due to stromal edema) related to the duration of the diseases [20, 24, 29, 30]. Conversely, the randomized trial of Duggan et al. [28], which involved 25 patients per group, found significant correlations between postoperative posterior HOAs and postoperative BCVA, and not between anterior HOAs and BCVA. These results need to be confirmed by studies on larger cohorts of patients. Limitations of our study are the retrospective design and the lack of preoperative aberrometric evaluation, that may add information about the preoperative severity of the disease.

Contrast sensitivity has been reported to be better after DMEK than after DSAEK, probably due to the asymmetry of the DSAEK graft or due to the stroma-to-stroma interface irregularities [31, 32]; furthermore, the contrast sensitivity after DMEK in phakic eyes was comparable to healthy eyes in previous studies [31, 32]. To our knowledge, no studies comparing contrast sensitivity between UT-DSAEK and DMEK have been published up till now. Our results in pseudophakic patients show that DMEK outperformed UT-DSAEK especially in mesopic conditions and at intermediate spatial frequencies; in both groups contrast sensitivity values were at the lower limit or below the age-standardized reference threshold of normal phakic patients. The most appropriate reference group would have been composed of pseudophakic controls, but contrast sensitivity values of pseudophakic controls measured using the Optec 6500 vision tester have not yet been published.

Finally, the satisfaction questionnaire showed that although patients were highly satisfied with both procedures, more than half of them preferred DMEK and reported more comfortable and quicker postoperative recovery. Although they were highly satisfied with their vision in both eyes, about one third of patients reported excellent vision only in DMEK eyes.

## Conclusions

In conclusion, according to our results, DMEK and UT-DSAEK showed no evidence of difference in terms of postoperative BCVA although DMEK had a better performance in terms of contrast sensitivity, posterior corneal aberrations and overall patient satisfaction. Moreover, our study confirms that the measurement of high-contrast visual acuity alone is an insufficient indicator of the subjective and objective visual performance of patients who underwent EK for endothelial disfunction. Visual outcome after EK may depend not only on the BCVA, which in our study did not differ significantly between the two techniques but may also be related to other parameters such as the thickness of the transplanted graft, corneal aberrations and contrast sensitivity. These factors, together with the speed of postoperative recovery may influence the overall patient satisfaction. Further studies on a larger number of patients are needed to confirm our results and to better analyze differences between UT- or NT-DSAEK and DMEK outcomes.

## Data Availability

The datasets used and analyzed during the current study are available from the corresponding author on reasonable request.
